# Empowering pro-environmental behavior in tourists through digital media: the influence of eco-guilt and empathy with nature

**DOI:** 10.3389/fpsyg.2024.1387817

**Published:** 2024-05-09

**Authors:** Xin Chen, Zhen-feng Cheng, Hui-juan Yang

**Affiliations:** ^1^College of Landscape Architecture and Tourism, Hebei Agricultural University, Baoding, China; ^2^Business School, Guangzhou College of Technology and Business, Guangzhou, China

**Keywords:** digital media information sharing, presence, eco-guilt, empathy with nature, tourists’ pro-environmental behavioral intention

## Abstract

In the digital economy era, leveraging digital media to foster tourists’ pro-environmental behavioral intention (TPEBI) has become crucial in the field of sustainable tourism. While existing studies have mainly focused on the driving mechanism of TPEBI within physical tourism contexts, the correlation between digital media information sharing and TPEBI remains unclear. Our study employs the cognitive-affective-conative framework to construct a theoretical model, considering eco-guilt and empathy with nature as mediating variables. It aims to explore the influencing mechanism of destination environmental information sharing through digital media on TPEBI from a presence perspective. Thereby, two scenario experiments were designed: Study 1 examined the impact of different formats of destination environmental threat information presentation on digital media on the sense of presence, while Study 2 explored the influencing mechanism of presence on TPEBI based on the conclusions of Study 1. Results indicate that (1) vivid and visible presentation formats of destination environmental threat information on digital media enhance individuals’ sense of presence; (2) sense of presence positively influences TPEBI; and (3) eco-guilt and empathy with nature mediate between presence and TPEBI. These findings not only contribute to theoretical and empirical research on digital media information sharing in sustainable tourism but also offer guidance for governments and tourism destinations to effectively stimulate TPEBI through digital media, achieve the sustainable development of destinations.

## 1 Introduction

The ecological environment stands as a cornerstone for the sustainable development of tourism destinations. However, the exponential surge in tourist arrivals has exacerbated the damage inflicted by tourism activities on destination ecosystems, posing significant challenges to sustainable development (Lee et al., 2021). Hence, it becomes paramount to educate tourists about their responsibilities and foster responsible behavior throughout their travels. Typically, tourism unfolds in unique environments and within constrained timeframes. The influence of daily moral norms on tourists may be limited, while the repercussions of violating social norms are relatively minor, often leading to uncivilized behavior (Zhang et al., 2019; Li and Chen, 2021). In recent years, media exposure has highlighted instances of improper conduct such as littering, trampling vegetation, and defacing landmarks by tourists in destinations. Consequently, discerning the primary factors influencing tourists’ pro-environmental behavioral intention (TPEBI) has emerged as a pivotal endeavor for the sustainable development of tourism destinations (Wang G. Q. et al., 2023).

Existing studies on the influencing mechanism of TPEBI primarily concentrate on the field tourism context, emphasizing aspects such as travel experience (Lin and Lee, 2020; Lee and Jan, 2023), destination psychological ownership (Qiu et al., 2022), and environmental commitment (Tang et al., 2022) as antecedent variables. However, they often overlook factors pertinent to the digital virtual context, hindering the widespread dissemination of environmental education. In light of the rapid proliferation of the Internet and intelligent terminal devices, individuals now possess the ability to share information regarding environmental threats in tourism destinations on digital media platforms at any time and from any location (Meng et al., 2023). This trend has the potential to draw greater attention to destination environmental issues. Nevertheless, the specific types of environmental information presentation formats (such as text-based, image-based, or video-based) that are most effective in capturing individuals’ attention toward ecological concerns in tourism destinations and subsequently stimulating their TPEBI remain unclear. Addressing these pertinent issues is imperative.

Several studies have indicated a close relationship between the presentation format of information in virtual environments and the sense of presence (Verhagen et al., 2014). The sense of presence, as an individual’s sensory experience within a media environment, reflects the extent to which individuals perceives themselves in the virtual setting (Ou et al., 2014). With the rapid advancements in mobile Internet and digital technology, the sense of presence finds widespread application in areas such as live-streamed shopping, information dissemination, and online education (Xu et al., 2021; Yao and Jia, 2021; Guo et al., 2023). Vonkeman et al. (2017) found that in online environments, the more vivid and interactive the presentation format of media information, the higher the sense of presence experienced by individuals. Furthermore, presence plays a crucial role in understanding attitudes and behaviors in virtual environments. Scholars have extensively explored the relationship between presence and individual behavioral decision-making, yielding significant insights. For example, Chen (2023) confirmed the positive impact of virtual presence on consumers’ online shopping intentions using the Stimulus-Organism-Response paradigm. Additionally, Yao and Jia (2021) explored the influence of presence on impulse travel intentions within the digital media context. It is evident that the sense of presence plays a pivotal role in mediating the relationship between the presentation format of media information and user behavioral intentions. However, the precise correlation mechanism of presence between digital media environmental information sharing and TPEBI remains elusive.

In light of this, our study constructs a mediation model based on the cognitive-affective-conative framework, incorporating eco-guilt and empathy with nature (affective factor). It aims to explore the influencing mechanism of destination environmental information sharing through digital media on TPEBI from the perspective of presence (cognitive factor). The main contributions of our study are as follows: (1) Examining the differences in the impact of various environmental threat information presentation formats via digital media on presence, which enriches our understanding of the formation mechanism of presence. (2) Clarifying that digital media serves as an effective pathway to stimulate TPEBI, which expands research on the driving mechanism of TPEBI from the traditional field tourism context to the digital virtual context. (3) Revealing the influencing mechanism of presence on TPEBI, which expands the explanatory power of presence to the domain of responsible tourism behavior, offering a fresh perspective for comprehending the relationship between digital media information sharing and individual behavior decision-making. (4) Verifying the mediating effect of eco-guilt and empathy with nature between presence and TPEBI, which provides a new theoretical framework for studying the influencing mechanism between presence and individual behavioral intention.

## 2 Literature review

### 2.1 Digital media information sharing

With the rapid advancement of digital technology and the widespread use of instant communication tools, various digital media platforms (such as Facebook, WeChat, Weibo, etc.) have permeated every aspect of social life, profoundly influencing and reshaping people’s consumption patterns, communication habits, collaborative efforts, and other behaviors (Liu et al., 2024). In the realm of tourism, digital media plays a pivotal role, subtly shaping and influencing tourists’ behavioral decisions (Yao and Jia, 2021). Existing studies on the impact of digital media information sharing on tourists predominantly focus on its effects on travel intentions (Yao and Jia, 2021; Hussain et al., 2024), tourism experiences (Jamshidi et al., 2023), destination image (Liu et al., 2024), with more attention devoted to analyzing the features of the shared information (Jamshidi et al., 2023). However, in reality, digital media information encompasses various presentation formats such as text, images, and videos, each potentially exerting different influences on tourists (Yao and Jia, 2021). Therefore, our study seeks to explore the disparities in the impact of different environmental information presentation formats on individual attitudes and behavioral decisions within the context of digital media.

### 2.2 Presence

The concept of presence originates from telepresence, denoting the extent to which individuals perceive reality within a virtual environment (Lombard and Jones, 2007). When an individual’s perception of the virtual environment surpasses that of the physical environment, a sense of presence ensues (Pelet et al., 2017). Scholars often categorize presence into two dimensions: spatial presence and social presence. Spatial presence, also referred to as physical presence, pertains to the authenticity of an individual’s perception of the physical environment constructed by virtual information, indicating a sense of immersion. Whereas, social presence relates to individuals’ perception of interaction within a virtual environment, emphasizing a feeling of companionship (Ou et al., 2014). In this study, presence primarily denotes the immersive feeling individuals experience when browsing destination environmental information on digital media, thereby aligning with spatial presence. As individuals’ perception of the authenticity of the media environment, presence holds significant implications for understanding their perception of media information and subsequent behavioral decision-making. Scholars have highlighted that within the context of tourism live-streamed shopping, factors such as information quality and source credibility can enhance individuals’ sense of presence, thereby stimulating their willingness to continue viewing (Sang et al., 2023). Similarly, when individuals browse tourism information shared via social media, they also experience a sense of presence, with higher perceived presence correlating with a greater likelihood of impulse travel intentions (Yao and Jia, 2021). However, whether individuals experience a sense of presence when perusing destination environmental information through digital media, and whether this resultant presence can further stimulate TPEBI, remain unresolved questions.

### 2.3 Tourists’ pro-environmental behavioral intention

Tourists’ pro-environmental behavior refers to actions undertaken by tourists within the tourism environment that contribute to promoting environmental sustainable development (Lee et al., 2013). Previous studies have demonstrated that TPEBI plays a pivotal role in enhancing environmental quality and fostering the sustainable development of tourism destinations (He et al., 2022). Consequently, understanding the driving mechanisms behind TPEBI has become a critical topic in sustainable tourism research. Within the tourism context, tourists often prioritize hedonic experiences and may overlook the environmental impact of their actions, making it challenging for them to voluntarily assume responsibility for environmental protection (Liu et al., 2021). Additionally, the unconventional nature of tourism environments weakens the influence of moral norms on tourists, diminishing their sense of responsibility during travel (Li and Chen, 2021). Consequently, instances of irresponsible behavior such as littering and scribbling are prevalent, resulting in direct or indirect adverse effects on tourist destinations (Cheng and Chen, 2022). Given this backdrop, stimulating TPEBI has become a pressing issue. While existing research has predominantly explored the influencing mechanisms of TPEBI within the field tourism context, scant attention has been paid to factors associated with the digital virtual context. With the proliferation of mobile Internet and digital technology, the influence of digital media on individual attitudes and behaviors has become increasingly significant. Therefore, it is imperative to investigate the substantial impact of digital media on TPEBI.

### 2.4 Eco-guilt

Guilt, characterized by a negative emotional response triggered by an individual’s recognition that their behavior violates social or moral norms and leads to negative consequences for society or others, Tangney and Dearing (2002) often motivates prosocial actions (Dahl et al., 2003; Lunardo and Saintives, 2018). According to cognitive dissonance theory, guilt arises from the discrepancy between one’s actions and moral standards, prompting individuals to engage in compensatory behaviors to alleviate the resulting psychological discomfort (Festinger, 1957; Theotokis and Manganari, 2015). Given the significant role of guilt in stimulating prosocial behavior, scholars have extensively investigated its relationship with various formats of positive conduct. For example, Hassan and Rahman (2023) discovered that guilt can deter unethical consumption behaviors. Similarly, Wang and Wu (2016) revealed that emotions like guilt prompt individuals to undertake compensatory actions, fostering a willingness to engage in sustainable consumption, particularly for energy-saving products. In recent years, amidst escalating concerns over issues such as global warming and environmental degradation, researchers have begun to explore the integration of guilt into the realm of environmental behavior. Some studies indicate that eco-guilt, as a kind of negative emotion arising when individuals perceive their responsibility for environmental consequences, can spur environmental protection actions, with its impact often surpassing that of positive emotions (Moore and Yang, 2020; Shipley and van Riper, 2022). While scholars have extensively discussed the relationship between eco-guilt and pro-environmental behaviors, there has been comparatively less focus on the driving mechanism of eco-guilt on TPEBI within the tourism context (Bahja and Hancer, 2021). Given the escalating prominence of environmental issues in tourist destinations and the pivotal role of tourists in shaping the sustainable development of these locales, it becomes imperative to explore the influencing mechanism of eco-guilt on TPEBI.

### 2.5 Empathy with nature

Empathy refers to the ability to put oneself in the other person’s position to identify with and understand another person’s emotions, thoughts, and experiences, essentially putting oneself in their shoes (Hogan, 1969). As ecological and environmental issues have become increasingly prominent, Tam (2013) expanded the scope of empathy to encompass the relationship between humans and nature, introducing the notion of empathy with nature. This concept suggests that empathy with nature entails an individual’s ability to empathize with the situation in nature. Individuals with a strong empathic connection to nature are better equipped to recognize and comprehend environmental challenges faced by nature, thereby fostering their sense of environmental responsibility (Liu, 2023). Empathy with nature not only enables individuals to gain profound insights into various environmental issues but also strengthens the emotional bond between humans and nature, motivating individuals to pay attention to and address environmental problems (Tam, 2013). Numerous studies have empirically demonstrated the relationship between empathy with nature and individual pro-environmental behaviors. For example, Wang L. T. et al. (2023) argue that individuals who empathize with nature are more inclined to take effective action to address environmental issues. Similarly, Ienna et al. (2022) found that individuals with a higher capacity to empathize with nature exhibit more positive environmental attitudes and are more willing to engage in pro-environmental behaviors. Therefore, our study contends that empathy with nature serves as a crucial perspective for investigating the driving factors of pro-environmental behavior, warranting empirical analysis of the relationship between empathy with nature and pro-environmental behavior.

## 3 Research hypotheses

### 3.1 Digital media information sharing and presence

Some scholars have noted that the formats of information presentation in digital media, including text, images, and videos, are closely linked to the sense of presence, with each form having a distinct impact on individuals’ presence (Yao and Jia, 2021). Generally, media information that is more vivid and interactive tends to facilitate a stronger sense of presence (Vonkeman et al., 2017). Yao and Jia (2021) highlighted that compared to text-based information, both image-based and video-based information are more likely to evoke a sense of presence in individuals. Similarly, Yu et al. (2017) found that presentation formats with enhanced visibility and interactivity can lead online consumers to experience a heightened sense of presence, thereby influencing their recommendation and purchase intentions. Verhagen et al. (2014), focusing on the online shopping context, confirmed that different product presentation formats exert varying impacts on presence, noting that virtual mirrors induce a higher sense of presence compared to 360-spin rotation and static images. These findings suggest that information with enhanced visibility and vividness tends to augment an individual’s sense of presence. Therefore, based on the above discussions, our study posits the following hypothesis:

*H1:* Video-based information presented by digital media evokes a higher sense of presence compared to image-based information, and the sense of presence induced by image-based information is higher than that induced by text-based information.

### 3.2 Presence and tourists’ pro-environmental behavioral intention

Presence, defined as the extent to which individuals perceive reality within virtual environments, significantly influences individual attitudes and behavioral decisions (Ou et al., 2014). In recent years, the academic community has increasingly focused on the relationship between presence and individual behavioral intention within the realms of virtual reality and digital technology. Sang et al. (2023) discovered that heightened presence experienced while viewing tourism live-streamed shopping content correlates with a greater likelihood of viewers continuing to watch. Similarly, Yao and Jia (2021) observed that stronger presence when browsing tourism information on social media corresponds to a greater impulse to travel. Scholars have also investigated the relationship between presence and individual behavioral intention in the online shopping context. For instance, Xu et al. (2021) found that the sense of presence induced by individuals watching online live-streamed shopping positively influences their purchase intention. Additionally, Dong et al. (2023) highlighted the significance of presence in live e-commerce, where it serves as a crucial incentive for consumers to experience flow and develop impulse consumption intentions. Building on this literature, our study posits that a higher sense of presence experienced by individuals when browsing destination environmental threat information through digital media facilitates a deeper understanding of the destination’s ecological environment, thereby stimulating TPEBI. Therefore, the following hypothesis is proposed:

*H2:* Presence positively influences TPEBI.

### 3.3 Mediating role of eco-guilt

According to the cognitive appraisal theory (Lazarus, 1991), individuals engage in cognitive evaluation in response to external stimuli, leading to emotional responses and subsequent coping behaviors, thereby forming an “evaluation-emotion-behavioral response” path relationship. According to this theory, when individuals browse information concerning threats to the ecological environment of tourism destinations via digital media, they undergo cognitive evaluation of the immersive experience, subsequently experiencing feelings of guilt regarding the ecological environment. This guilt then activates their pro-environmental behavioral intentions, thus facilitating the “evaluation-emotion-behavioral response” pathway. Existing studies have also proved the mediating role of affective emotions between individual cognitive evaluation (perceived tourism impact) and behavioral response (support for tourism) on the basis of cognitive appraisal theory (Munanura et al., 2023). Additionally, according to the stimulus-organism-response theory, individuals’ emotions are influenced by external stimuli, leading to corresponding behavioral responses (Mehrabian and Russell, 1974). In this study, the sense of presence induced by individuals browsing destination environmental threat information through digital media serves as an external stimulus. This sense of presence further triggers individuals’ eco-guilt (organism), subsequently stimulating their pro-environmental behavioral intentions (behavioral response). Compared to individuals’ direct observation of environmental issues in the field, the immersive experience generated by viewing media information tends to evoke stronger feelings of guilt, worry, and anxiety regarding environmental issues, thus reducing the psychological distance between individuals and the ecological environment (Wang et al., 2017), and consequently stimulating their pro-environmental behavioral intentions (Lange and Dewitte, 2022). Based on the aforementioned discussion, our study proposes the following hypotheses:

*H3:* Presence positively affects eco-guilt.

*H4:* Eco-guilt positively affects TPEBI.

*H5:* Eco-guilt plays a mediating role between presence and TPEBI.

### 3.4 Mediating role of empathy with nature

The empathy-altruism hypothesis proposed by Batson (1987) provides a theoretical basis for revealing the intermediary transmission mechanism between presence and pro-environmental behavior. According to the theory, individuals encountering information regarding destination environmental threats through digital media are likely to experience emotional resonance with the environmental pollution and ecological damage faced by those destinations. This emotional resonance, in turn, activates their willingness to engage in behaviors aimed at solving environmental problems. Moreover, following the cognitive-affective-conative theoretical framework (Mischel and Shoda, 1995), the sense of presence (cognitive factor) generated when individuals browse destination environmental threat information through digital media can promote their empathy with nature (emotional factor), and consequently stimulate their pro-environmental behavioral intentions. Therefore, the cognitive-affective-conative framework provides a theoretical basis for elucidating the mediating mechanism between presence and TPEBI. Empirical analyses in existing studies have corroborated the mediating role of empathy between presence and individual prosocial behaviors (Kandaurova and Lee, 2019; Lee and Li, 2023). Therefore, our study posits that the presence experienced by individuals when browsing destination environmental threat information through digital media can elicit empathy toward nature. This empathy with nature, in turn, further stimulates their willingness to engage in pro-environmental behavior. Based on these premises, the study proposes the following hypotheses:

*H6:* Presence positively affects empathy with nature.

*H7:* Empathy with nature positively affects TPEBI.

*H8:* Empathy with nature plays a mediating role between presence and TPEBI.

**Figure 1:**
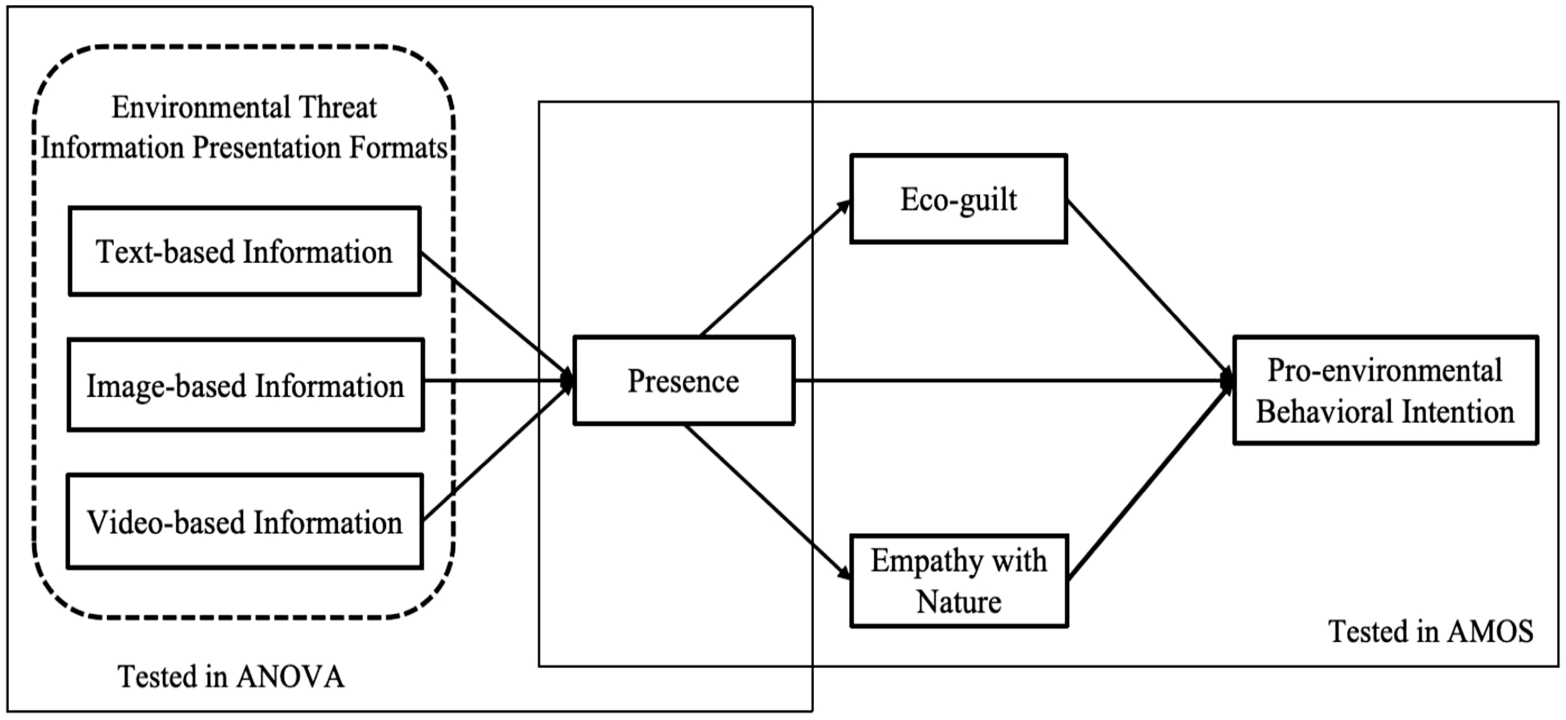
Theoretical model.

In summary, drawing from a review of literature and practical research, our study constructs a theoretical model (as shown in Figure 1) to empirically investigate the influencing mechanism of destination environmental threat information on digital media on TPEBI.

## 4 Study 1

### 4.1 Design

In Study 1, a scenario experiment was conducted to examine the impact of various formats of environmental threat information presented via digital media on individuals’ sense of presence. Our study selects Chinese digital media users as the research object. There are a large number of digital media users in China, and their behaviors and attitudes are deeply influenced by digital media. In this context, it is more necessary and urgent to explore how to enhance Chinese users’ pro-environmental behavior through digital media. Participants were recruited through Credamo, a prominent Chinese online research platform, and were offered incentives as a token of appreciation for their participation. To reflect the primary methods through which digital media presents destination environmental information, the study selected three presentation formats-text, images, and videos-depicting environmental threats on Weibo, a widely influential digital media platform in China. To eliminate the bias stemming from participants’ preconceived notions about the destination, the experiment’s design concealed the destination’s name. The manipulation materials for the independent variables consisted of three groups: Text-based information group: Participants were exposed to written descriptions of the destination’s environmental threats. Image-based information group: Participants viewed visual representations illustrating the ecological threats faced by the destination. Video-based information group: Participants watched videos depicting the environmental threats present at the destination.

### 4.2 Pre-test and measures

To assess the effectiveness of the independent variable manipulation and the validity of measurement items, a pre-test was conducted. The pre-test was mainly carried out in the classroom. Three classes were selected and the experimental materials were randomly allocated among the classes. In this pre-test, 72 participants were randomly assigned to one of three experimental scenarios. They were then instructed to read the corresponding stimulus material, complete measures of scenario authenticity and presence, and provide demographic information. The measurement item for scenario authenticity was adapted from Dabholkar (1994), with the specific item being “in real life, the above scenario may happen.” The measurement items of presence were adapted from Xu et al. (2021) and Yin et al. (2023), comprising four items. These items assessed the participants’ feelings when browsing destination environmental threat information through digital media, including statements like “I have a feeling of being there,” “I feel that the tourism destination is in front of me,” “I feel that I am in the real world created by digital media,” and “I can really perceive the existence of environmental threats in the destination.” A total of 68 valid questionnaires were collected from the pre-experiment (21 in G_text_, 23 in G_image_ and 24 in G_video_). Among the participants, 41.1% were male and 58.9% were female. And they were mainly between 18 and 24. One-way analysis of variance (ANOVA) was performed, revealing that the degree of presence reported by participants exposed to video-based information was significantly higher than that reported by those exposed to image-based and text-based information (M_text_ = 2.83, SD = 0.48; M_image_ = 3.37, SD = 0.37; M_video_ = 4.21, SD = 0.47; *p* < 0.001). This result indicates successful manipulation of the independent variable. Furthermore, reliability analysis demonstrated that Cronbach’s α value for the presence measurement items was 0.887, exceeding the threshold of 0.7, indicating good reliability. Thus, these measures were deemed suitable for use in the formal experiments.

### 4.3 Procedure

Through the Chinese online research platform Credamo, 180 participants were recruited to partake in the formal experiment, with the participants randomly assigned to three groups (60 in G_text_, 60 in G_image_ and 60 in G_video_). Prior to the commencement of the experiment, the research team thoroughly explained the experiment requirements and relevant instructions to the participants, emphasizing the anonymity of the process. Participants were then asked to complete a questionnaire based on their genuine reactions to browsing the experimental material. The experimental stimulus materials, the authenticity test of the scenario, and the measurement methods for the sense of presence were consistent with those utilized in the pre-test. To ensure the experiment’s alignment with the context of digital media usage, questionnaires were administered online. Additionally, a question pertaining to the ecological environment information of the destination featured in the experimental materials was incorporated into the questionnaire. Only questionnaires with complete browsing of the experimental materials and accurately filled responses were considered valid. Following the removal of incomplete questionnaires, 161 valid questionnaires were obtained (51 in G_text_, 56 in G_image_, and 54 in G_video_). Among the participants, 45.8% were male and 54.2% were female; 73.1% were aged 35 or below, 18.7% were between 36 and 45, and 8.2% were over 45. Furthermore, the majority of participants held a college degree or higher (94.3%).

### 4.4 Results

**Figure 2:**
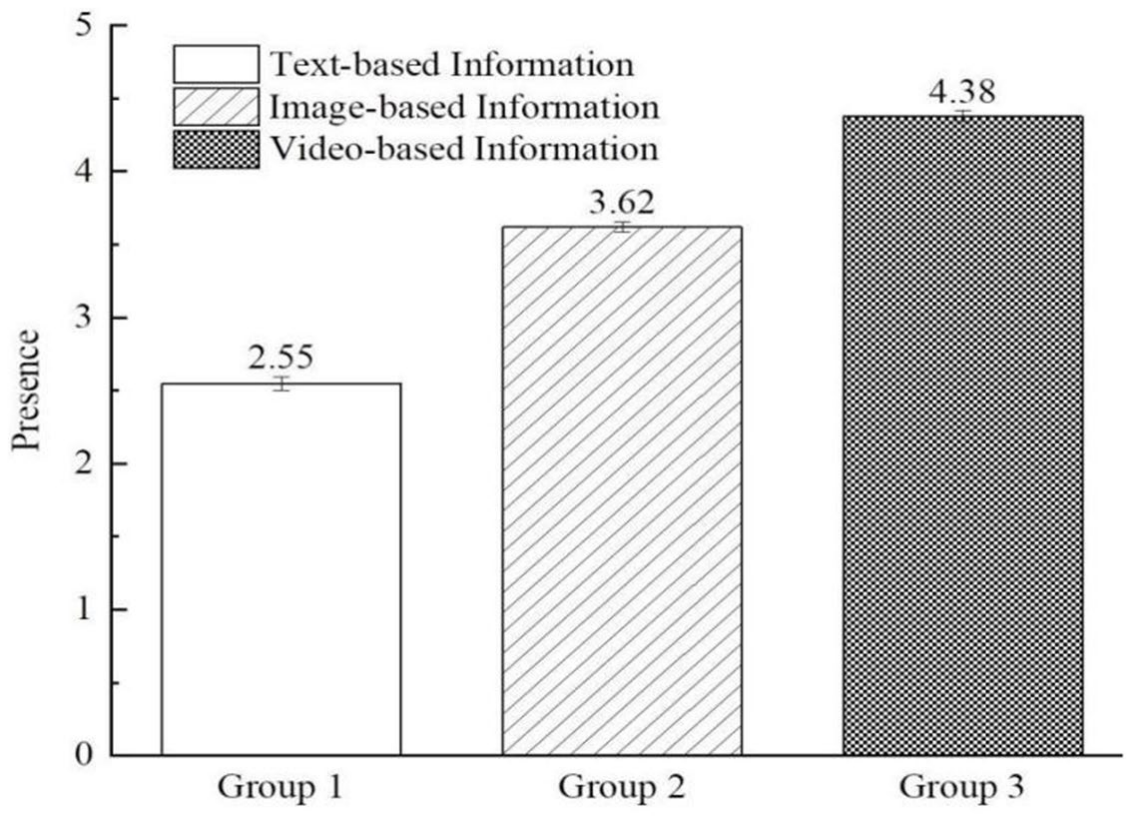
The impact of digital media information presentation formats on presence (study 1).

The results of the scenario authenticity test indicated that more than 95 percent (98.4 percent) of participants, perceived the scenarios as realistic. Additionally, the reliability analysis revealed a Cronbach’s α coefficient of 0.848, greater than 0.7, for the measurement items, indicating good reliability. In this study, one-way ANOVA analysis was conducted to assess the impact of the three types of information presentation on the sense of presence. The ANOVA analysis treated presence as the dependent variable and the presentation form of digital media information as the independent variable. The results unveiled significant differences in the influence of the three formats of information presentation on the sense of presence [*F*_(2, 158)_ = 534.03, *p* < 0.001]. As depicted in Figure 2, the presence reported by participants in the video group was significantly higher than that in the image group (M_image_ = 3.62, SD = 0.25; M_video_ = 4.38, SD = 0.26; *p* < 0.001) and text group (M_text_ = 2.55, SD = 0.34; M_video_ = 4.38, SD = 0.26; *p* < 0.001). Moreover, the presence in the image group was significantly higher than that in the text group (M_text_ = 2.55, SD = 0.34; M_image_ = 3.62, SD = 0.25; *p* < 0.001). These findings indicate that compared to text and image presentations, conveying environmental threats information through short videos can significantly enhance individuals’ sense of immersion. Thus, Hypothesis H1 is supported.

## 5 Study 2

### 5.1 Design and measures

Study 2, aims to investigate the mechanism through which presence influences TPEBI. The experimental materials and design are the same as those in Study 1. A total of 366 participants were recruited through the Credamo online research platform and randomly assigned to one of three experimental groups (G_text_, G_image_, and G_video_). Participants were then asked to browse the experimental materials and complete various measurement items, including scenario authenticity, presence, eco-guilt, empathy with nature, and pro-environmental behavioral intention, and demographic information. The measurement items for scenario authenticity and presence were consistent with those used in Study 1. Additionally, the measurement items for eco-guilt, empathy with nature, and pro-environmental behavioral intention were adapted from existing scales. To ensure the applicability of the English scale in the Chinese context, our study adopted the back-translation method to carry out translational work. For eco-guilt, three measurement items were utilized, adapted from Bahja and Hancer (2021) and Onwezen et al. (2013). Specific items are: “I will feel guilty,” “I will feel ashamed,” and “I will feel uneasy if I do not pay attention to ecological protection when traveling.” Empathy with nature was measured using three items adapted from scales designed by Li et al. (2019) and Liu (2023). These items are: “I can perceive the pain suffered by the animals and plants in the destination,” “I can imagine the difficult situation of the animals and plants in the destination.” and “I care and sympathize with the animals and plants in the destination.” Pro-environmental behavioral intention was assessed using three items, mainly referring to Cheng and Chen (2022) and Zhao et al. (2020). Specific items are: “I will abide by relevant policies and not destroy the ecological environment of the destination,” “I will prevent the behavior of damaging the ecological environment of the destination,” and “I will participate in activities to protect the ecological environment of the destination.” After removing invalid questionnaires (with confusing or non-serious answers), 329 valid questionnaires were obtained, with sample sizes of 106, 109, and 114 in the text, image, and video groups, respectively. The demographic distribution included 43.2% male and 56.8% female participants, with 67.3% aged 35 or below, 23.6% between 36 and 45, and 9.1% over 45. The majority of participants had a college degree or higher (91.8%). On observation of the demographic characteristics it was found that it mostly comprised young participants, who are more familiar with digital media platforms and have strong acceptance of new technologies.Therefore, the characteristics of the sample in our study are in line with the reality that the majority of digital media users in China are young people, indicating that the survey sample is representative.

### 5.2 Reliability and validity analysis

The reliability of the measurement tools was assessed using Cronbach’s *α* coefficient. The results indicated that the Cronbach’s *α* values for presence, eco-guilt, empathy with nature, and pro-environmental behavioral intention were 0.919, 0.892, 0.890, and 0.904, respectively. All of these values exceeded the threshold of 0.7, demonstrating good reliability for each scale.

The validity of the measurement scale was assessed by examining both convergent validity and discriminant validity through confirmatory factor analysis using AMOS 28.0 software. Table 1 presents the results. The standardized factor loadings for all measured items ranged from 0.806 to 0.894, with composite reliability (CR) values ranging from 0.891 to 0.920, all exceeding the threshold of 0.7. Additionally, the average variance extracted (AVE) values ranged from 0.732 to 0.760, all surpassing the recommended threshold of 0.5. These findings indicate strong convergent validity for all scales.

**Table 1:** Reliability and convergent validity test.

Variable	Item	Significance				Reliability	Convergent Validity	
		Unstd.	S.E.	Z	Std.	Cronbach’s α	CR	AVE
Presence (PR)	PR1	1.000			0.806	0.919	0.920	0.742
	PR2	1.247	0.067	18.750	0.883			
	PR3	1.189	0.064	18.512	0.875			
	PR4	1.220	0.065	18.669	0.880			
Eco-guilt (EG)	EG1	1.000			0.859	0.892	0.892	0.734
	EG2	0.998	0.052	19.049	0.875			
	EG3	1.001	0.055	18.122	0.836			
Empathy with nature (EN)	EN1	1.000			0.846	0.890	0.891	0.732
	EN2	1.089	0.058	18.853	0.879			
	EN3	0.920	0.051	18.008	0.842			
Tourists’ Pro-environmental Behavioral Intention(TPEBI)	TPEBI1	1.000			0.894	0.904	0.905	0.760
	TPEBI2	0.896	0.043	21.036	0.864			
	TPEBI3	0.874	0.042	20.752	0.857			

Unstd., unstandardized factor loading; S.E., standard error; Std: standardized factor loading; AVE, average variance extracted; CR, composite reliability.

The discriminant validity of the measurement scale was assessed by comparing the square root value of AVE of each variable with the correlation coefficient between that variable and other variables. The results are presented in Table 2. It was found that the square root value of AVE for each variable exceeded the correlation coefficient between variables, demonstrating satisfactory discriminant validity for all scales.

**Table 2:** Discriminant validity test.

Variable	PR	EG	EN	TPEBI
PR	0.861			
EG	0.345	0.857		
EN	0.403	0.343	0.855	
TPEBI	0.536	0.473	0.550	0.872

The on-diagonal items are square roots of AVE; off-diagonal items are correlation coefficients between the variables; PR, presence; EG, eco-guilt; EN, empathy with nature; TPEBI, tourists’ pro-environmental behavioral intention.

### 5.3 Hypotheses testing

#### 5.3.1 Relationship between the destination environmental threat information on digital media and the sense of presence

**Figure 3:**
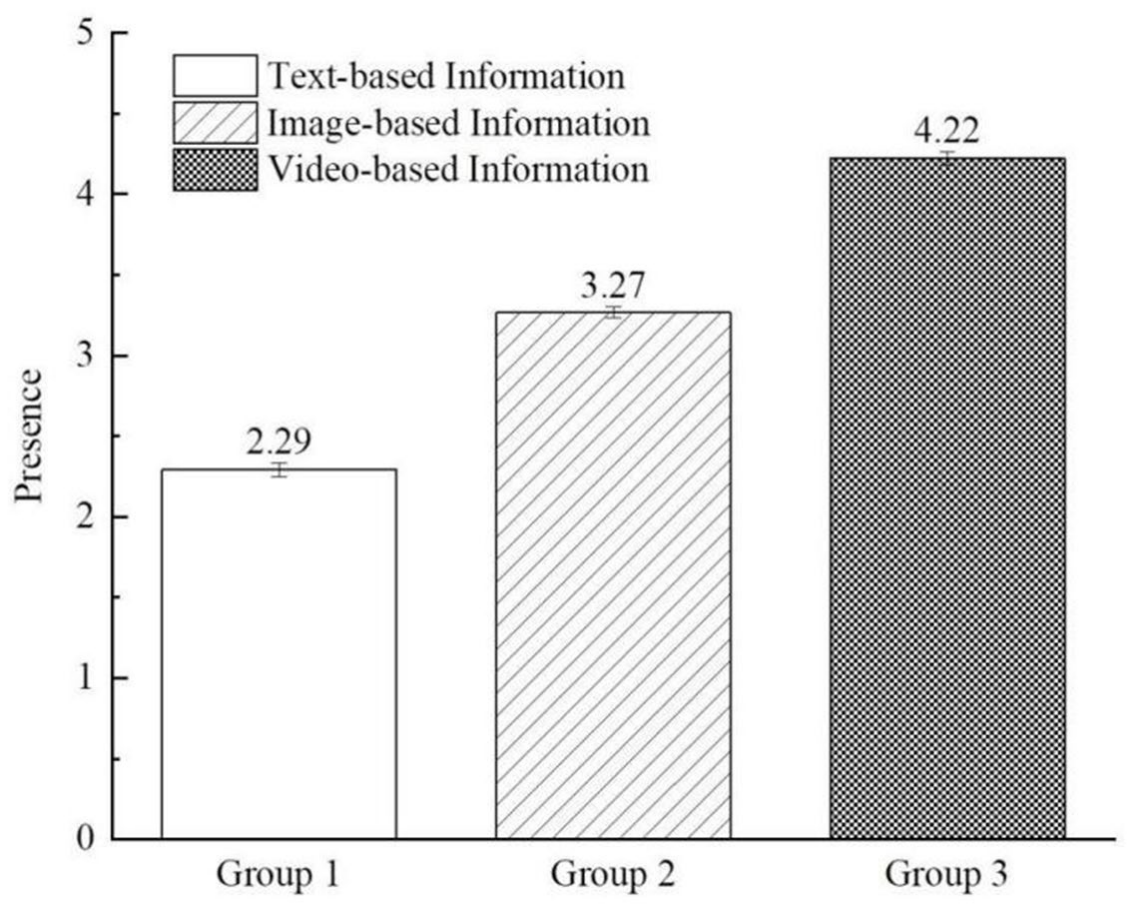
The impact of digital media information presentation formats on presence (study 2).

To further corroborate the findings of Study 1, we conducted a one-way ANOVA analysis to examine the difference in the impact of different presentation formats of destination environmental threat information on digital media on individual sense of presence. The results revealed significant differences in the influence of various information presentation formats on individual presence [*F*_(2, 326)_ = 592.325, *p* < 0.001]. As depicted in Figure 3, the sense of presence in the video group significantly surpassed that in the image and text groups (M_text_ = 2.29, SD = 0.45; M_image_ = 3.27, SD = 0.36; M_video_ = 4.22, SD = 0.43; *p* < 0.001), thereby further affirming Hypothesis H1. Furthermore, our study further examined the differences in the effects of distinct information presentation formats on eco-guilt, empathy with nature, and pro-environmental behavioral intention. The results demonstrated significant differences in the levels of eco-guilt [*F*_(2, 326)_ = 20.575, *p* < 0.001], empathy with nature [*F*_(2, 326)_ = 26.637, *p* < 0.001], and pro-environmental behavioral intention [*F*_(2, 326)_ = 51.606, *p* < 0.001] induced by varied information presentation formats, with video-based information eliciting higher responses compared to image-based and text-based information.

#### 5.3.2 Relationship between presence and tourists’ pro-environmental behavioral intention

Before conducting the main effect test, we used AMOS 28.0 software to analyze the fit index of the structural model. The results indicated a good fit degree (X^2^/DF = 0.990, GFI = 0.974, CFI = 0.959, NFI = 0.981, RMSEA = 0.011), suggesting that the structural model was suitable for further analysis. The results of main effect test based on the structural equation model showed that the sense of presence exhibited a positive promoting effect on TPEBI [*β* = 0.411, BC 95% CI = (0.284, 0.552), *p* < 0.001], supporting Hypothesis H2. Additionally, presence demonstrated significant positive effects on both eco-guilt [*β* = 0.488, BC 95% CI = (0.338, 0.641), *p* < 0.01] and empathy with nature [*β* = 0.525, BC 95% CI = (0.393, 0.665), *p* < 0.001]. Both eco-guilt [*β* = 0.276, BC 95% CI = (0.174, 0.376), *p* < 0.001] and empathy with nature [*β* = 0.412, BC 95% CI = (0.299, 0.527), *p* < 0.01] were found to further stimulate TPEBI. These findings suggest that eco-guilt and empathy with nature may mediate the relationship between presence and TPEBI. Therefore, Hypotheses H3, H4, H5, H6, H7, and H8 are preliminarily supported.

#### 5.3.3 Mediating effect test

The bootstrapping method was employed to further verify the mediating effect of eco-guilt and empathy with nature between presence and TPEBI, with 5,000 iterations and a confidence level set to 95%. The results indicate that presence indirectly influences TPEBI through eco-guilt [*β* = 0.135, BC 95% CI = (0.078, 0.211), *p* < 0.01] and empathy with nature [*β* = 0.216, BC 95% CI = (0.148, 0.309), *p* < 0.001] (refer to Table 3). This finding suggests that eco-guilt and empathy with nature indeed play a mediating role between presence and TPEBI. Consequently, Hypotheses H3, H4, H5, H6, H7, and H8 are further corroborated. Considering the direct positive promoting effect of presence on TPEBI [*β* = 0.411, BC 95% CI = (0.284, 0.552), *p* < 0.001], it can be inferred that eco-guilt and empathy with nature partially mediate the relationship between presence and TPEBI.

**Table 3:** Indirect and direct effects analysis.

Paths	Estimates	Product of Coefficient		Bootstrapping	
				Bias-Corrected 95% CI	
		S.E.	*Z*	Lower Limit	Upper Limit
Indirect effects					
PR -- > EG-- > TPEBI	0.135	0.033	4.091	0.078	0.211
PR -- > EN-- > TPEBI	0.216	0.041	5.268	0.148	0.309
Direct effects					
PR-- > EG	0.488	0.076	6.441	0.338	0.641
EG-- > TPEBI	0.276	0.052	5.333	0.174	0.376
PR-- > EN	0.525	0.071	7.415	0.393	0.665
EN-- > TPEBI	0.412	0.058	7.103	0.299	0.527
PR -- > TPEBI	0.411	0.073	5.633	0.284	0.552

PR, presence; EG, eco-guilt; EN, empathy with nature; TPEBI, tourists’ pro-environmental behavioral intention.

## 6 Discussion and conclusion

Based on the perspective of the sense of presence, our study, through a combination of scenario experiments and questionnaire surveys, has examined the difference in the impact of various formats of destination environmental threat information presented by digital media on individual presence. Additionally, it has elucidated the influencing pathway of presence on TPEBI. The specific conclusions drawn are as follows:

First, different formats of destination environmental threat information presented by digital media exhibit significant differences in their impact on individuals’ sense of presence. Video-based information elicits the highest level of presence, followed by image-based information, while text-based information has the lowest impact. This finding underscores the notion that the more immersive the presentation form of environmental threat information in digital media, the more likely it is to engender a sense of immersion in individuals. This result further corroborates the research findings of Yao and Jia (2021) regarding the relationship between information sharing and presence in digital media. Second, presence emerges as a pivotal inducement to trigger TPEBI, highlighting its crucial role in driving individuals to engage in pro-environmental behavior. Specifically, when individuals peruse information concerning threats to the destination’s ecological environment on digital media, the sense of presence serves to diminish the psychological distance between them and the destination’s ecological environment. Consequently, they become more inclined to care about and address environmental issues, ultimately fostering a willingness to protect the ecological environment of destinations. Third, presence not only directly fosters TPEBI but also indirectly influences it through eco-guilt and empathy with nature. Specifically, information regarding threats to the destination’s ecological environment, presented with vividness and visibility on digital media, can evoke individuals’ immersive experiences, subsequently stimulating their emotional responses of eco-guilt and empathy with nature. This, in turn, enables individuals to empathize with the vulnerability of the destination’s ecological environment, propelling them toward pro-environmental behavior. It is evident that eco-guilt and empathy with nature serve as significant emotional driving mechanisms effectively fostering individuals’ engagement in pro-environmental behavior.

### 6.1 Theoretical implications

First, amidst the escalating environmental challenges in tourist destinations, the exploration of the driving forces behind TPEBI has garnered considerable attention. Existing studies predominantly delve into the formation mechanisms of TPEBI within the context of on-site tourism experiences, encompassing factors such as destination image, tourism encounters, and environmental commitment, among others (He et al., 2018; Tang et al., 2022; Lee and Jan, 2023). However, scant attention has been paid to the influence of virtual environments, such as digital media, on TPEBI (Han et al., 2023). Building upon existing research, our study empirically scrutinized the presentation formats of destination environmental threat information on digital media and the ensuing influence of presence on TPEBI. This conclusion broadened the scope of inquiry established by Shang et al. (2021) regarding the nexus between information dissemination on digital platforms and individual behavioral intentions, thereby further enriching the literature on the determinants of TPEBI (Tu and Ma, 2022; Li et al., 2023a).

Second, existing studies examining the impact of presence on individual behavioral decision-making primarily concentrate on contexts such as live-streamed shopping and online education (Xu et al., 2021; Guo et al., 2023), with limited discussion on the influencing mechanism of presence on individual pro-environmental behavioral intention from an ecological perspective. Our study extends the observed effects of presence to the realm of individual pro-environmental behavioral intention, offering a novel lens for comprehending the interplay between digital media information dissemination and individual behavioral decision-making. In doing so, it broadens the scope of the influence of presence, thus contributing to a deeper understanding of the relationship between digital media and human behavior (Amin et al., 2021; Shu et al., 2023; Li et al., 2023b).

Finally, existing research on the correlation mechanism between presence and individual behavioral decision-making is relatively scarce. Our study attempts to explore a novel mediating mechanism in the formation of TPEBI by investigating the mediating effect of eco-guilt and empathy with nature between presence and TPEBI. This enriches the research findings of Shu et al. (2023) that presence can indirectly influence individuals’ behavioral decisions by shaping their emotional experiences. By providing a new analytical framework for understanding the influencing path between presence and behavioral intention, our study contributes to the broader theoretical discussion on the correlation mechanism between digital media use and individual behavioral decision-making (Rajarathinam et al., 2022; Choi and Noh, 2023).

### 6.2 Practical implications

First, the accurate and transparent dissemination of information regarding destination environmental threats through digital media serves as a pivotal means to foster TPEBI. As environmental challenges in tourism destinations escalate, stakeholders such as governments and tourism authorities must leverage platforms like WeChat, Weibo, and TikTok to conduct comprehensive environmental education initiatives. By enhancing public awareness of environmental issues and the detrimental effects of irresponsible behavior on destination ecosystems, individuals are more likely to engage in pro-environmental actions. Additionally, it is imperative for governments, tourism authorities, and media outlets to effectively communicate and supervise the dissemination of information regarding threats to destination environments through digital channels, thus highlighting the consequences of environmental degradation. Furthermore, empowering individuals with the necessary skills to participate in environmental conservation efforts can bolster their willingness to address and mitigate environmental challenges in destinations, fostering a culture of environmentally responsible behavior.

Additionally, our study underscores the significant impact of different presentation formats of destination environmental threat information on individuals’ sense of presence and pro-environmental behavioral intentions. Notably, the more immersive and visually compelling the digital media presentations are, the greater the individual’s sense of presence and inclination toward pro-environmental action. These findings offer valuable theoretical insights for governmental bodies, tourism authorities, and other stakeholders seeking to leverage digital media for environmental advocacy. When disseminating information about the environmental challenges facing tourism destinations through digital platforms, it is essential for relevant entities to enhance management and intervention in information presentation formats to ensure the efficacy of environmental messaging and educational efforts. Additionally, the public should be encouraged to share high-quality digital media content aimed at promoting environmental protection in destinations, thereby bolstering their awareness of environmental issues and sense of responsibility. Specifically, information regarding threats to the ecological integrity of tourism destinations should be conveyed through digital media using engaging formats such as videos and images, with the integration of cutting-edge technologies like 3D, virtual reality (VR), and augmented reality (AR) to enhance visual immersion. Moreover, efforts should be made to guide the public in sharing content that highlights the environmental damage incurred by destinations, utilizing short videos or images, while implementing effective management and monitoring mechanisms to filter out any harmful or misleading content. Furthermore, digital technologies should be harnessed to authentically depict the environmental challenges faced by destinations, vividly illustrating the adverse consequences of environmental degradation in a visually compelling manner.

Furthermore, our study reveals that the sense of presence indirectly influences TPEBI through eco-guilt, highlighting the importance of leveraging emotional factor of eco-guilt and stimulate responsible pro-environmental behavior among tourists. On the one hand, with the rapid development of digital technologies such as VR, AR and artificial intelligence, government and tourism destinations should employ real-time monitoring systems to track threats and ecological damage to tourism destinations. By leveraging digital platforms, scenes depicting environmental degradation and its severe consequences can be effectively conveyed to tourists, evoking a sense of guilt about the state of the destination’s environment and subsequently motivating them toward pro-environment behavioral intentions. On the other hand, relevant agencies can also stimulate tourists’ eco-guilt through various communication channels, including public lectures, educational campaigns, and on-site exhibitions. For instance, issues pertaining to ecological damage and environmental pollution, along with their detrimental effects, can be disseminated via popular digital media platforms such as WeChat, Weibo, and TikTok.

Finally, our study found that empathy with nature plays a mediating role between presence and TPEBI, highlighting the importance of enhancing tourists’ empathy with nature to foster pro-environmental behavior. To this end, various strategies can be implemented to deepen tourists’ connection with the natural environment. For example, relevant authorities should leverage digital media platforms to share the current state of the ecological environment in destinations, allowing the public to gain a deeper understanding of the fragility of the ecological environment. Additionally, engaging and informative activities focused on human-nature relationships can be popularized, utilizing creative short videos to underscore the importance of harmonious coexistence between humans and nature. Moreover, anthropomorphic representations of the tourist landscape can be designed to evoke empathy among tourists. Utilizing VR, AR, and other digital technologies, immersive experiences can be created to vividly depict the plight of animals and plants in tourism destinations, thereby enhancing tourists’ empathy with nature.

### 6.3 Limitations and future research directions

First, it’s important to acknowledge that our study primarily relies on self-report. Considering that pro-environmental behavioral intention is a positive pro-social behavioral intention, the participants may have a tendency to meet social expectations when filling out questionnaires, thus affecting the authenticity of research conclusions to a certain extent. Future studies could incorporate a wider range of data collection methods to enhance the robustness of the measurement tools and improve the overall scientific rigor of the research. Second, our study focuses solely on examining the determinants of TPEBI, without delving into the factors influencing the translation of pro-environmental behavioral intention into actual pro-environmental behavior. Future studies can further explore the mechanism between TPEBI and their pro-environmental behavior, so as to further expand the research findings. Finally, our study only discusses the mediating role of eco-guilt and empathy-with nature between presence and TPEBI. Future studies can explore other mediating mechanisms and boundary conditions between presence and TPEBI.

## Data availability statement

The raw data supporting the conclusions of this article will be made available by the authors, without undue reservation.

## Ethics statement

Ethical review and approval was not required for the study on human participants in accordance with the local legislation and institutional requirements. Written informed consent from the [patients/ participants OR patients/participants legal guardian/next of kin] was not required to participate in this study in accordance with the national legislation and the institutional requirements.

## Author contributions

XC: Conceptualization, Data curation, Formal analysis, Methodology, Software, Supervision, Visualization, Writing – original draft, Funding acquisition. Z-fC: Conceptualization, Data curation, Funding acquisition, Investigation, Project administration, Resources, Supervision, Writing – review & editing. H-jY: Data curation, Investigation, Methodology, Project administration, Resources, Supervision, Visualization, Writing – review & editing.
